# Genome‐Wide CRISPR Screen Reveals PIK3CA Inhibition Enhances Lipid Nanoparticle‐Mediated siRNA Delivery

**DOI:** 10.1002/advs.202517617

**Published:** 2025-12-07

**Authors:** Wenhan Wang, Kangfu Chen, Zongjie Wang

**Affiliations:** ^1^ Chan Zuckerberg Biohub Chicago Chicago IL 60607 USA; ^2^ Department of Biomedical Engineering McCormick School of Engineering Northwestern University Evanston IL 60208 USA; ^3^ School of Integrated Circuits and Electronics Beijing Institute of Technology Beijing 100081 P. R. China

**Keywords:** cancer treatment, CRISPR, functional genomics, inflammatory diseases, lipid nanoparticles, siRNA

## Abstract

Lipid nanoparticles (LNPs) are useful carriers for therapeutic siRNA delivery, yet their clinical efficacy remains constrained by insufficient cellular uptake. Here, using a genome‐wide CRISPR knockout screen, multiple genetic modulators of LNP uptake is uncovered, with PIK3CA emerging as a top druggable target. Pharmacologic inhibition of PIK3CA with BAY1082439 – a clinically evaluated small molecule – significantly enhances LNP uptake, siRNA delivery, and gene silencing across diverse epithelial cancer cell lines in vitro. Co‐administration of BAY1082439 with siRNA‐loaded LNPs also better suppressed tumor growth and reduced liver inflammation in vivo, respectively. These findings establish PIK3CA inhibition as a broadly applicable strategy to boost LNP‐mediated RNA interference and highlight the promise of combining functional genomics with nanomaterials to advance RNA‐based therapeutics.

## Graphical abstract

A genome‐wide CRISPR screen and identifies PIK3CA as a key regulator of lipid nanoparticle (LNP) uptake is performed. Modulating PIK3CA with small‐molecule inhibitor BAY1082439 enhanced LNP uptake, siRNA delivery, and gene silencing in a variety of epithelial cells, and improved therapeutic outcomes in vivo. The work presents PIK3CA inhibition as a promising strategy to boost LNP‐based RNA therapeutics.

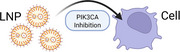

## 1 Introduction

Nanoparticles (NPs), particularly lipid nanoparticles (LNPs), have transformed the delivery of therapeutic nucleic acids such as mRNA and siRNA.^[^ ^1^, ^2^, ^3^ ^]^ The clinical success of LNPs in mRNA‐based COVID‐19 vaccines has sparked widespread interest in leveraging NPs for treating cancer,^[^ ^4^, ^5^ ^]^ inflammatory conditions,^[^ ^6^ ^]^ and infectious diseases.^[^ ^7^ ^]^ However, despite these advances, the in vivo delivery efficiency of NPs remains suboptimal, often leading to insufficient therapeutic efficacy in clinical trials.^[^ ^8^ ^]^ This warrants the need for continued efforts to improve NP delivery in vivo.

A seminal study identified two major barriers to effective in vivo NP delivery: tissue specificity at the systemic level and cellular uptake efficiency within target tissues.^[^ ^9^ ^]^ Indeed, this study examined the fate of cancer‐targeting NPs administered through intravenous injection and found that only 0.7% of the administered NPs ended up in tumor tissues. Within the tumor environment, only 0.2% of the intratumoral NPs properly entered the intracellular domain of cancer cells.^[^ ^9^ ^]^ These findings highlight the dual challenge of enhancing both tissue‐level targeting and cellular‐level uptake.

To address the challenge of cellular uptake efficiency, a substantial body of research has focused on optimizing NPs designs. These efforts have spanned a variety of approaches, such as modifying surfaces with uptake‐promoting ligands,^[^ ^10^ ^]^ as well as tuning particle size and charge.^[^ ^11^ ^]^ While these strategies have shown promise, their impact remains limited. Increasingly, studies are shifting focus toward understanding the role of recipient cell biology in NP uptake. These efforts reveal that delivery outcomes are shaped not only by NP properties but also by cellular factors such as cell type,^[^ ^12^ ^]^ cell cycle stage,^[^ ^13^ ^]^ and adhesion dynamics.^[^ ^14^ ^]^ Such intrinsic biological features profoundly influence NP internalization and downstream RNA delivery. However, our understanding of how these cellular factors govern uptake remains limited. This underscores the need for comprehensive investigations that correlate individual cellular factors with delivery efficiency.

The advent of CRISPR technology has enabled researchers to systematically perturb individual genes across the entire human genome using libraries of guide RNAs (gRNAs) and study the resulting cellular phenotypes in specific biological processes.^[^ ^15^ ^]^ This approach, commonly referred to as CRISPR screening, has facilitated comprehensive genome‐wide analyses linking genetic perturbations to phenotypes of interest – such as the emergence of highly potent tumor‐killing immune cells^[^ ^16^ ^]^ or drug‐resistant cancer cells.^[^ ^17^ ^]^ Its widespread adoption has significantly advanced our comprehensive understanding of key biological processes, including cancer metastasis,^[^ ^18^ ^]^ tumor proliferation,^[^ ^19^ ^]^ and immune cytokine secretion,^[^ ^20^ ^]^ in a systematic and less biased manner.

**Figure 1:**
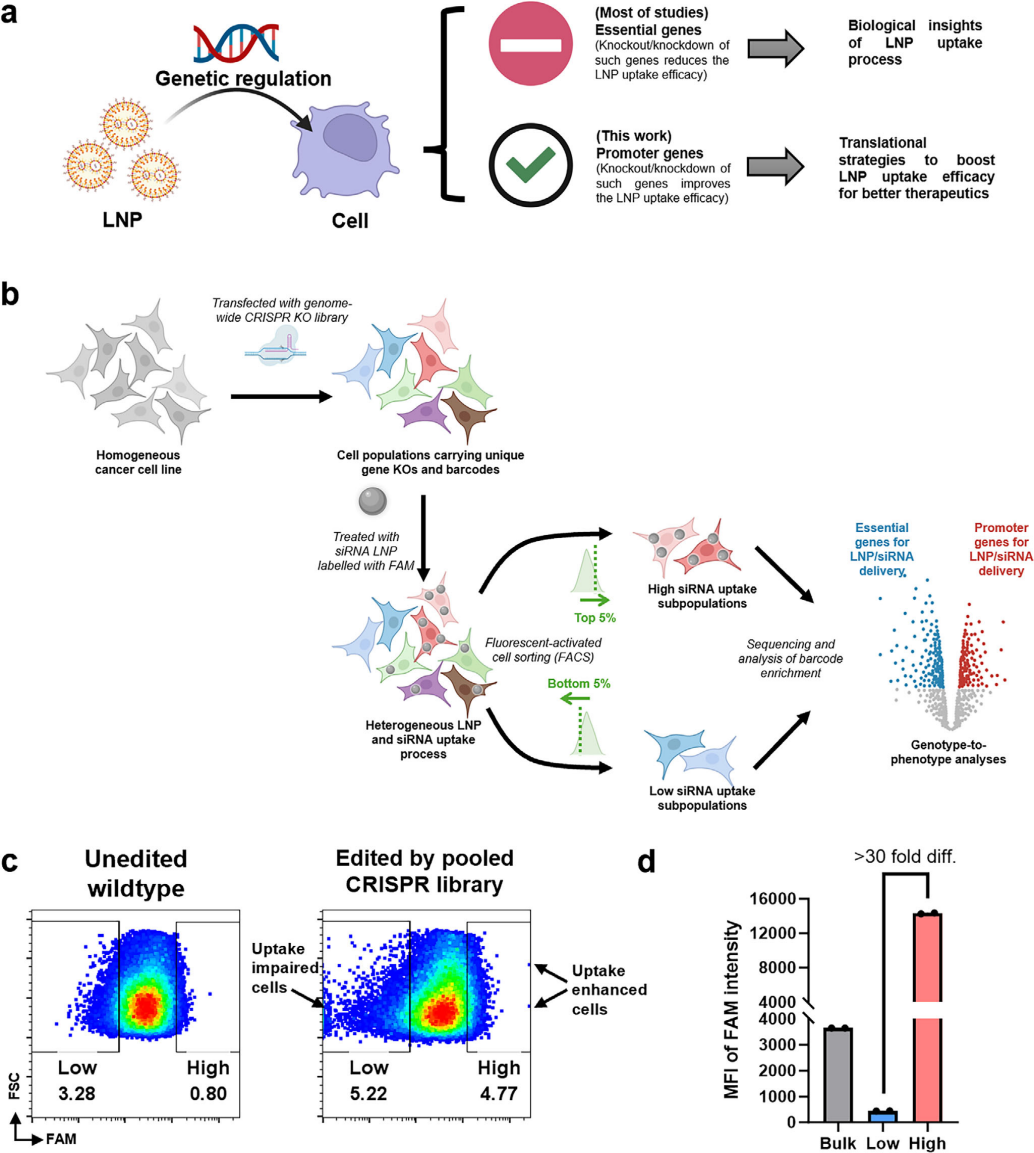
Discovering promoter genes to enhance LNP delivery efficacy. A) Conceptual framework for screening promoter genes involved in LNP delivery. While most published studies have focused on essential genes required for LNP uptake, identifying promoter genes – those that enhance uptake – may offer a more direct strategy to improve clinical translation and therapeutic outcomes. B) Overview of the pooled CRISPR screening workflow. A genome‐wide CRISPR knockout library was introduced into SW480 colorectal cancer cells, which were subsequently treated with FAM‐labeled siRNA‐loaded LNPs. Cells were then sorted by FACS into high‐ and low‐FAM intensity populations. C) Genome editing perturbed LNP uptake dynamics, resulting in distinct cell subpopulations with markedly different uptake efficiencies. D) Quantification of FAM fluorescence intensity in sorted populations. The FAM‐high and FAM‐low groups exhibited a >30‐fold difference in median fluorescence intensity (*n* = 3).

In the past three years, CRISPR screening has been applied to investigate the genetic regulators of NP uptake,^[^ ^21^, ^22^, ^23^ ^]^ leading to the identification of critical pathways essential for effective NP delivery^[^ ^21^ ^]^ and downstream mRNA expression.^[^ ^23^ ^]^ However, these studies typically isolate cell subpopulations with poor NP uptake or mRNA expression from the bulk population, thereby highlighting only the pathways required for baseline delivery (**Figure** **1**a). Cell subpopulations with enhanced NP uptake are often overlooked, despite their high potential for improving therapeutic outcomes.

In this study, we conducted a genome‐wide CRISPR knockout screen in a cancerous epithelial cell line using siRNA‐loaded LNPs and isolated both high and low NP uptake subpopulations. While we validated known pathways essential for LNP uptake, we also identified a subset of gene knockouts that significantly enhanced LNP internalization (Figure 1a). Among these genes, PIK3CA stood out due to its strong effect and the availability of FDA‐approved small‐molecule inhibitors targeting this gene. We investigated the impact of co‐administering PIK3CA inhibitors during LNP treatment and observed a consistent increase in LNP uptake – up to 5‐fold – across multiple epithelial cell types. This enhanced uptake translated into improved gene silencing efficacy of the delivered siRNA.

Finally, we demonstrated that PIK3CA co‐administration boosts LNP uptake and therapeutic outcomes in vivo, using two biologically distinct in vivo settings – a malignant tumor model and a non‐malignant inflammatory model – selected specifically to represent fundamentally different disease contexts. These findings suggest that PIK3CA inhibition is a simple yet effective strategy to enhance the performance of LNP‐based siRNA therapies.

## 2 Results

### 2.1 Screen Workflow

We began the study by transfecting the SW480 cancer cell line with a genome‐wide lentiviral CRISPR knockout (KO) library (Figure 1b). Specifically, we used the Toronto KnockOut (TKO) library, which comprises 70948 guide RNAs (gRNAs) targeting 18053 protein‐coding genes in the human genome.^[^ ^24^ ^]^ The library was prepared in a single‐vector system that co‐expresses Cas9, a specific gRNA, and a puromycin resistance marker to facilitate gene editing and selection. Lentiviral transfection was performed at a carefully controlled multiplicity of infection (MOI) of 0.25–0.4 to minimize the likelihood of individual cells receiving more than one gRNA. Following puromycin selection, untransfected cells were largely eliminated, yielding a heterogeneous SW480 population with individual gene knockouts. To assess the quality of transfection, we performed next‐generation sequencing (NGS) and found that over 98% of gRNAs were represented by more than 10 sequencing reads (Figure S1, Supporting Information). This indicated that our CRISPR library preparation has low gRNA dropout and minimal bias in the CRISPR‐edited cell population prior to nanoparticle treatment.

CRISPR‐edited SW480 cell populations were treated with LNPs encapsulating fluorescently labeled siRNA (FAM‐siRNA) at a dose of 1 µg mL^−1^ for 24 h. These LNPs were formulated via standard microfluidic mixing using an MC3‐based formulation (see Figure S2, Supporting Information for characterization of their biophysical properties). After 24 hrs of treatment, a subset of cells was analyzed for FAM fluorescence using flow cytometry. Compared to the unedited parental population, CRISPR‐edited cells exhibited a broader distribution of FAM fluorescence intensity (Figures 1c; S3, Supporting Information), suggesting that certain gene knockouts significantly influenced LNP uptake. Based on this observation, we used fluorescence‐activated cell sorting (FACS) to isolate the top 5% and bottom 5% of the CRISPR‐edited population based on fluorescence intensity (Figure 1b). These sorted subpopulations demonstrated a striking ≈50‐fold difference in median fluorescence intensity (MFI) (Figure 1d), confirming substantial variability in LNP uptake efficiency. This result provides direct evidence that genetic perturbations can modulate cellular uptake of LNPs and establishes a functional link between gene regulation and nanoparticle uptake.

### 2.2 Analysis of Hits

**Figure 2:**
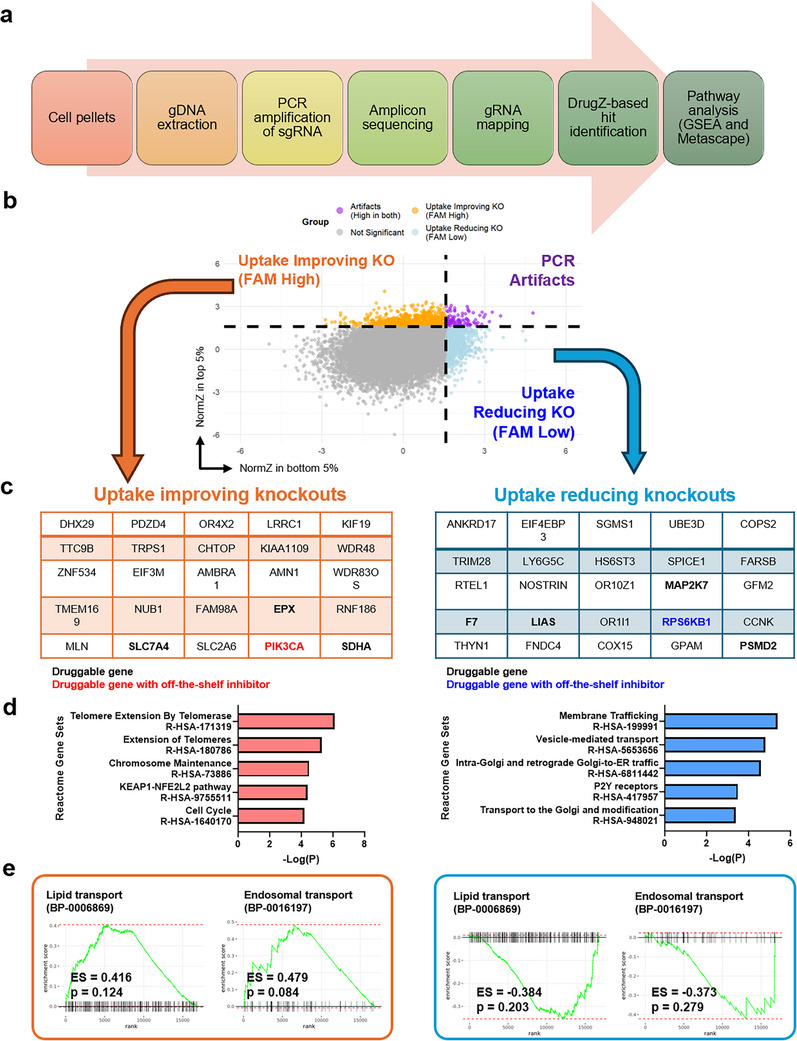
Identification of genetic regulators of LNP uptake through sequencing and bioinformatic analysis. A) Schematic of the sequencing and computational analysis workflow used to identify gene regulators influencing LNP uptake. B) NormZ scores for individual genes in FAM‐high and FAM‐low sorted populations, using the unsorted population as a reference. The NormZ score represents the degree of enrichment or depletion of each gene in the sorted populations. Genes with high positive NormZ scores are more enriched in a given population. Genes enriched in both FAM‐high and FAM‐low populations were excluded to minimize artifacts, such as PCR amplification bias. C) Top‐ranked genes based on NormZ scores. Druggable genes are highlighted in bold; those with readily available commercial inhibitors are further highlighted in bold with color. D) Pathway overrepresentation analysis of top‐ranked genes. Genes associated with reduced LNP uptake were significantly enriched in pathways known to be involved in LNP uptake and processing, such as membrane trafficking and vesicle transport. This validates the effectiveness of the conducted CRISPR screen. E) Gene set enrichment analysis (GSEA) of lipid transport and endosomal trafficking pathways, both critical for LNP uptake. The FAM‐high and FAM‐low subpopulations exhibited opposite enrichment trends, consistent with differential regulatory mechanisms of uptake efficiency.

Sorted subpopulations, along with unsorted cells collected on the same day, were used for downstream genomic DNA extraction, gRNA amplification, and sequencing (**Figure** **2**a). Raw sequencing reads were mapped to individual gRNA counts, and the drugZ algorithm was applied to identify differentially represented gRNAs between the sorted and unsorted populations, as recommended by the developers of the TKOv3 library.^[^ ^25^ ^]^ The primary output of drugZ is the NormZ score, which quantifies the relative enrichment or depletion of each gene. A positive NormZ score indicates that the corresponding gRNA is more abundant in the sorted population. For example, when comparing the top 5% of cells (high uptake) to the unsorted population, a high positive NormZ score suggests that KO of the associated gene enhances LNP uptake.

The full distribution of NormZ scores is shown in Figure S4 (Supporting Information), and the complete dataset is provided in the supplementary materials. We plotted the NormZ scores for both the top 5% (high uptake) and bottom 5% (low uptake) populations in Figure 2b. After filtering out gRNAs enriched in both populations – likely due to PCR amplification bias – we identified the top 25 gene KOs associated with increased or decreased LNP uptake (Figure 2c). Notably, ≈ 20% of these hits correspond to druggable genes, including *PIK3CA*, for which small‐molecule inhibitors are readily available. This facilitates downstream validation by enabling pharmacological suppression of these targets at the protein level.

To assess the biological relevance of our screen, we performed gene set enrichment analysis (GSEA) and overrepresentation analysis (ORA). Gene KOs associated with reduced uptake were significantly enriched in pathways related to membrane trafficking and vesicle transport (Figure 2d), consistent with known mechanisms governing NP internalization. Interestingly, pathway analysis revealed opposite trends in lipid transport and endosomal transport between the high‐ and low‐uptake populations (Figure 2e). The strong positive enrichment of lipid and endosomal transport in high‐uptake cells aligns well with the established mechanism for LNP internalization via lipid‐mediated endocytosis^[^ ^26^ ^]^ and subsequent endosomal escape for effective cargo release (e.g., siRNA).^[^ ^27^ ^]^

### 2.3 In Vitro Validation

**Figure 3:**
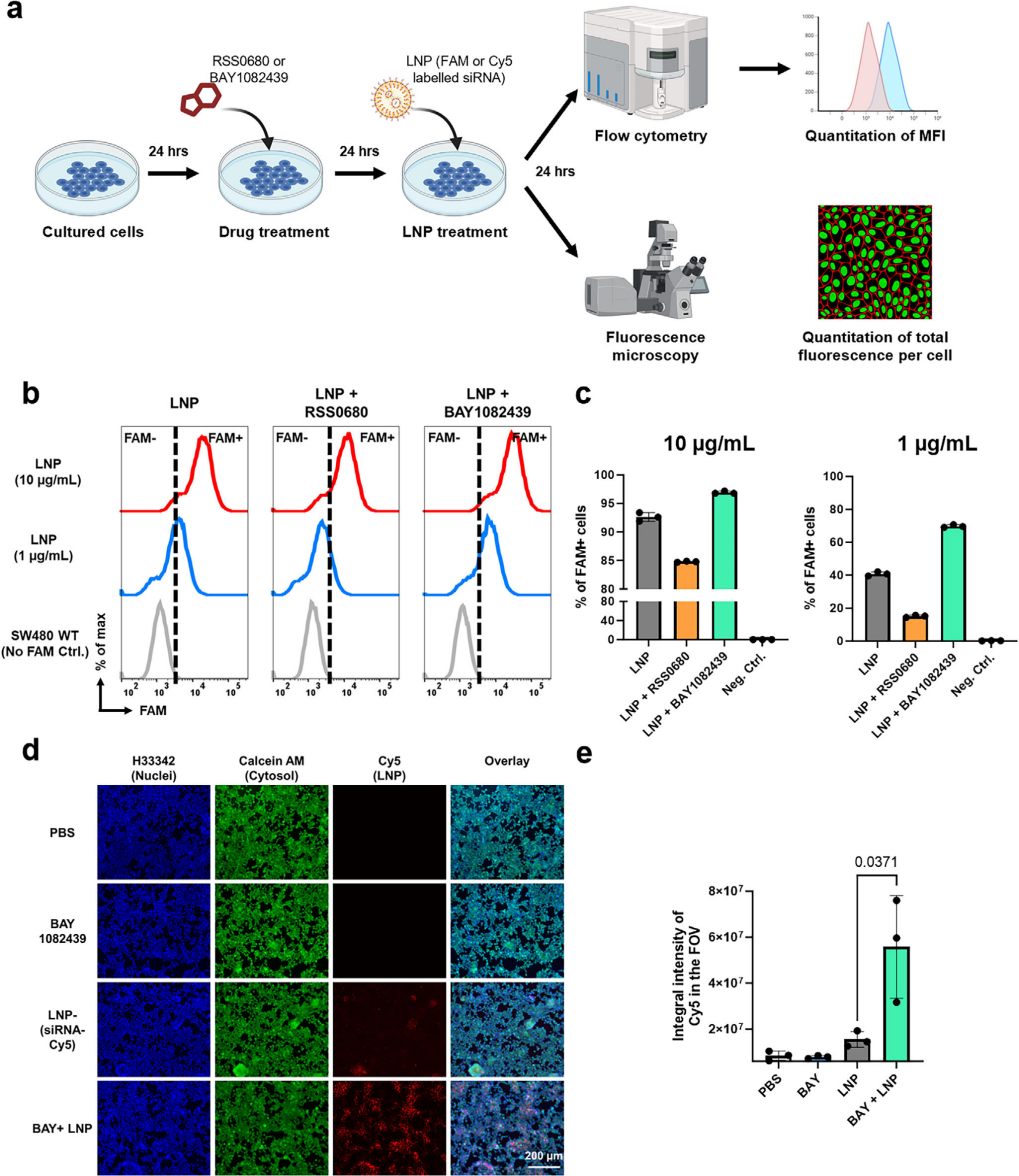
Functional validation of screen‐identified genetic regulators of LNP uptake. A) Schematic of the small‐molecule‐based validation workflow. SW480 cells were pretreated for 24 h with small‐molecule inhibitors targeting screen‐identified genes – RSS0680 (targeting RPS6KB1) and BAY1082439 (targeting PIK3CA). Cells were then treated with a consistent batch and dose of FAM‐ or Cy5‐labeled siRNA‐loaded LNPs. Uptake efficiency was assessed by fluorescence‐based quantification. B) Representative flow cytometry plots showing FAM‐labeled siRNA uptake 24 h post‐LNP treatment in cells with or without inhibitor treatment. C) Quantification of the percentage of FAM‐positive cells following treatment with each inhibitor (*n* = 3, unpaired t‐test).D) Representative fluorescence microscopy images of cells 24 h post‐treatment with Cy5‐labeled siRNA‐LNPs. E) Quantification of integrated Cy5 fluorescence intensity per field of view in treated samples (*n* = 3, unpaired t‐test).

To validate the validity of our screen hits, we selected one druggable gene from each uptake subpopulation: *PIK3CA* from the top 5% (uptake‐enhancing) population and *RPS6KB1* from the bottom 5% (uptake‐reducing) population. In addition to its druggability, *PIK3CA* was chosen for its central role in the functional network enrichment analysis performed using STRING, based on genes with *p* < 0.01 (Figure S5, Supporting Information). Both genes have well‐characterized small‐molecule inhibitors – BAY1082439 for PIK3CA^[^ ^28^ ^]^ and RSS0680 for RPS6KB1^[^ ^29^ ^]^ – with established pharmacological activity and pharmacokinetics. Based on literature‐reported dosing, we pre‐treated SW480 cells with BAY1082439 at 15 nm or RSS0680 at 100 nm for 24 h prior to LNP exposure (**Figure** **3**a). Following pharmacological suppression, cells were incubated with LNPs encapsulating fluorescently labeled negative control siRNA for an additional 24 h, and uptake was assessed via flow cytometry and/or fluorescence microscopy (Figure 3a).

Representative flow cytometry histograms for each treatment condition are shown in Figure 3b. As indicated by the dashed line, cells treated with RSS0680 exhibited reduced uptake of FAM‐labeled siRNA‐LNPs, whereas cells treated with BAY1082439 showed a marked increase in fluorescence intensity. To quantify these differences, we compared the percentage of FAM‐positive cells (Figure 3c) and the median fluorescence intensity (MFI) across treatment groups (Figure S6, Supporting Information). A consistent trend was observed. Yet, both small molecules – particularly BAY1082439 – have been previously characterized as anti‐cancer agents due to their ability to inhibit the growth of specific tumor cell lines.^[^ ^30^ ^]^ To ensure that the observed enhancement in LNP uptake was not an artifact of reduced cell number or viability due to growth inhibition, we performed fluorescence microscopy to assess cell density, viability, and nanoparticle uptake (Figure 3d). At the concentrations used, BAY1082439 did not show significant cytotoxicity in SW480 cells. This observation aligns with previous reports indicating that BAY1082439 is primarily effective in PTEN‐deficient cancer models^[^ ^31^ ^]^ and KRAS‐mutant cells are insensitive to BAY1082439 treatment.^[^ ^32^ ^]^ Importantly, from the microscopic images, we found that BAY1082439‐treated cells consistently displayed higher intracellular accumulation of LNPs, as visualized by Cy5‐labeled siRNA, with signal intensity reaching up to a 5‐fold increase compared to the LNP‐only group (Figure 3e).

Collectively, these results demonstrate that the capacity of cancer cells to internalize siRNA‐loaded LNPs is strongly influenced by their genetic profile. Moreover, druggable targets identified in our CRISPR screen, such as PIK3CA and RPS6KB1, can be effectively modulated with small‐molecule inhibitors to alter LNP uptake behavior. Besides, our preliminary validation data showed that inhibition of PIK3CA using BAY1082439 significantly enhanced LNP uptake in SW480 cells. This result is particularly encouraging for two reasons. First, BAY1082439 has demonstrated oral bioavailability in human patients,^[^ ^33^ ^]^ making it highly compatible with co‐administration alongside LNP‐based therapies. Second, according to data from the Human Protein Atlas,^[^ ^34^ ^]^ PIK3CA is widely expressed throughout the human body, with over 50% of organs exhibiting medium to high expression levels (Figure S7, Supporting Information). These observations suggest that PIK3CA inhibition could serve as a broadly applicable strategy to enhance LNP uptake and improve the therapeutic efficacy of siRNA delivery platforms.

**Figure 4:**
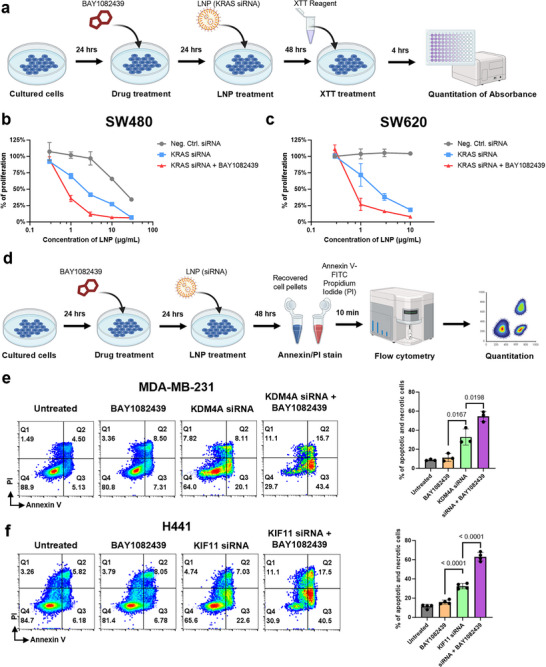
PIK3CA inhibition enhances the therapeutic efficacy of siRNA‐loaded LNPs in vitro. A) Experimental workflow for evaluating the combinatorial therapeutic effect of KRAS‐targeting siRNA and PIK3CA inhibition using BAY1082439. KRAS‐mutant‐dependent cancer cell lines SW480 and SW620 were treated with BAY1082439 and transfected with KRAS‐targeting siRNA. Cell proliferation was assessed using the XTT assay. B) Quantification of SW480 cell proliferation under various treatment conditions (LNPs with KRAS‐targeting siRNA ± BAY1082439, *n* = 3, unpaired t‐test). C) Quantification of SW620 cell proliferation under various treatment conditions (LNPs with KRAS‐targeting siRNA ± BAY1082439, *n* = 3, unpaired t‐test). D) Workflow for evaluating the therapeutic enhancement of siRNAs targeting essential genes in the presence or absence of PIK3CA inhibition. Based on previous literature, KDM4A knockdown induces apoptosis in MDA‐MB‐231 cells, while KIF11 silencing is known to cause apoptotic or necrotic cell death in H441 cells. E) Representative flow cytometry plots and quantification of apoptosis in MDA‐MB‐231 cells treated with the LNPs containing KDM4A‐targeting siRNA with or without BAY1082439 (*n* = 3, unpaired t‐test). F) Representative flow cytometry plots and quantification of apoptosis in H441 cells treated with the LNPs containing KIF11‐targeting siRNA with or without BAY1082439 (*n* = 3, unpaired t‐test).

To further evaluate the potential of PIK3CA inhibition, we conducted two sets of experiments: one to assess the functional efficacy of siRNA delivery (**Figure** **4**a–c), and another to examine the broader applicability of PIK3CA inhibition across different epithelial cancer cell types (Figure 4d–h). For the functional assessment, we delivered KRAS‐targeting siRNAs to SW480 and SW620 cells, both of which harbor the KRAS G12V mutation and rely heavily on KRAS expression for proliferation.^[^ ^35^ ^]^ Thus, the effectiveness of the delivered siRNA was evaluated by measuring growth inhibition in response to treatment (Figure 4b,c). Co‐treatment with the PIK3CA inhibitor BAY1082439 significantly enhanced the sensitivity of both cell lines to KRAS‐targeting siRNA, improving the growth‐inhibitory effect by up to 10‐fold. To evaluate broader applicability, we delivered KDM4A‐targeting siRNAs to MDA‐MB‐231 breast cancer cells^[^ ^36^ ^]^ and KIF11‐targeting siRNAs to H441 lung cancer cells.^[^ ^37^ ^]^ Inhibition of these genes were previously shown to induce apoptosis in these respective cell types.^[^ ^36^, ^37^ ^]^ Apoptotic and necrotic cell populations were quantified using Annexin V and propidium iodide (PI) staining (Figure 4d). Compared to LNP treatment alone, co‐administration of BAY1082439 significantly increased the apoptotic cell population – from 20.1% to 43.4% in MDA‐MB‐231 cells (Figure 4e), and from 22.6% to 40.5% in H441 cells (Figure 4f). Taken together, these results suggest that PIK3CA inhibition can consistently enhance the therapeutic efficacy of siRNA‐loaded LNPs across multiple epithelial cancer cell lines.

In addition to siRNA‐loaded LNPs, we evaluated luciferase mRNA‐loaded LNPs formulated with SM‐102, and ALC‐0315 lipids. Bioluminescence assays revealed a consistent increase in luciferase protein expression (Figure S8, Supporting Information). This suggests that the PIK3CA inhibition strategy is broadly applicable across diverse LNP formulations and RNA cargo types. Furthermore, siRNA‐mediated knockdown of PIK3CA in cells treated with mRNA‐loaded LNPs produced a comparable enhancement in LNP uptake (Figure S9, Supporting Information). This provides direct evidence that the stimulatory effect of BAY1082439 arises from specific inhibition of PIK3CA activity rather than off‐target effects. Finally, dose‐response analysis confirmed that 15 nm BAY1082439 represents the minimal concentration required to achieve maximal upregulation of LNP uptake in vitro (Figure S10, Supporting Information).

To investigate the underlying mechanism of PIK3CA inhibition on LNP uptake, we examined EGFP mRNA‐loaded LNPs containing a fluorescently tagged lipid (DiD). Flow cytometry analysis showed that cells subjected to PIK3CA inhibition exhibited significantly enhanced endocytic uptake, as indicated by increased DiD fluorescence (Figure S11a,b, Supporting Information). Indeed, PI3K signaling influences cytoskeletal dynamics and membrane ruffling.^[^ ^38^ ^]^ Thus, inhibition of PIK3CA may promote cytoskeleton‐driven uptake pathways, such as phagocytosis and macropinocytosis,^[^ ^39^ ^]^ which can facilitate increased LNP internalization. It is also noteworthy that PIK3CA has been reported to play a central role in regulating endosomal trafficking and maturation via phosphoinositide lipid signaling.^[^ ^40^, ^41^ ^]^ Its inhibition may delay the transition from early to late endosomes,^[^ ^41^ ^]^ thereby prolonging the residence time of LNPs within early endosomal compartments and increasing opportunities for endosomal escape. This hypothesis is indirectly supported by our observation that the EGFP‐to‐DiD fluorescence ratio modestly increased following PIK3CA inhibition (Figure S11b,c, Supporting Information).

### 2.4 In Vivo Validation

Motivated by its potent effects in vitro, we next evaluated whether PIK3CA inhibition could enhance the therapeutic efficacy of LNP‐mediated siRNA delivery in vivo. BAY1082439 has previously been investigated as an anti‐tumor agent in preclinical prostate cancer models and evaluated in a Phase I clinical trial (NCT01728311) to assess its safety and pharmacokinetics at doses ranging from 50 to 75 mg kg^−1^. Based on this precedent, we selected a dose of 50 mg kg^−1^ for our in vivo studies.

**Figure 5:**
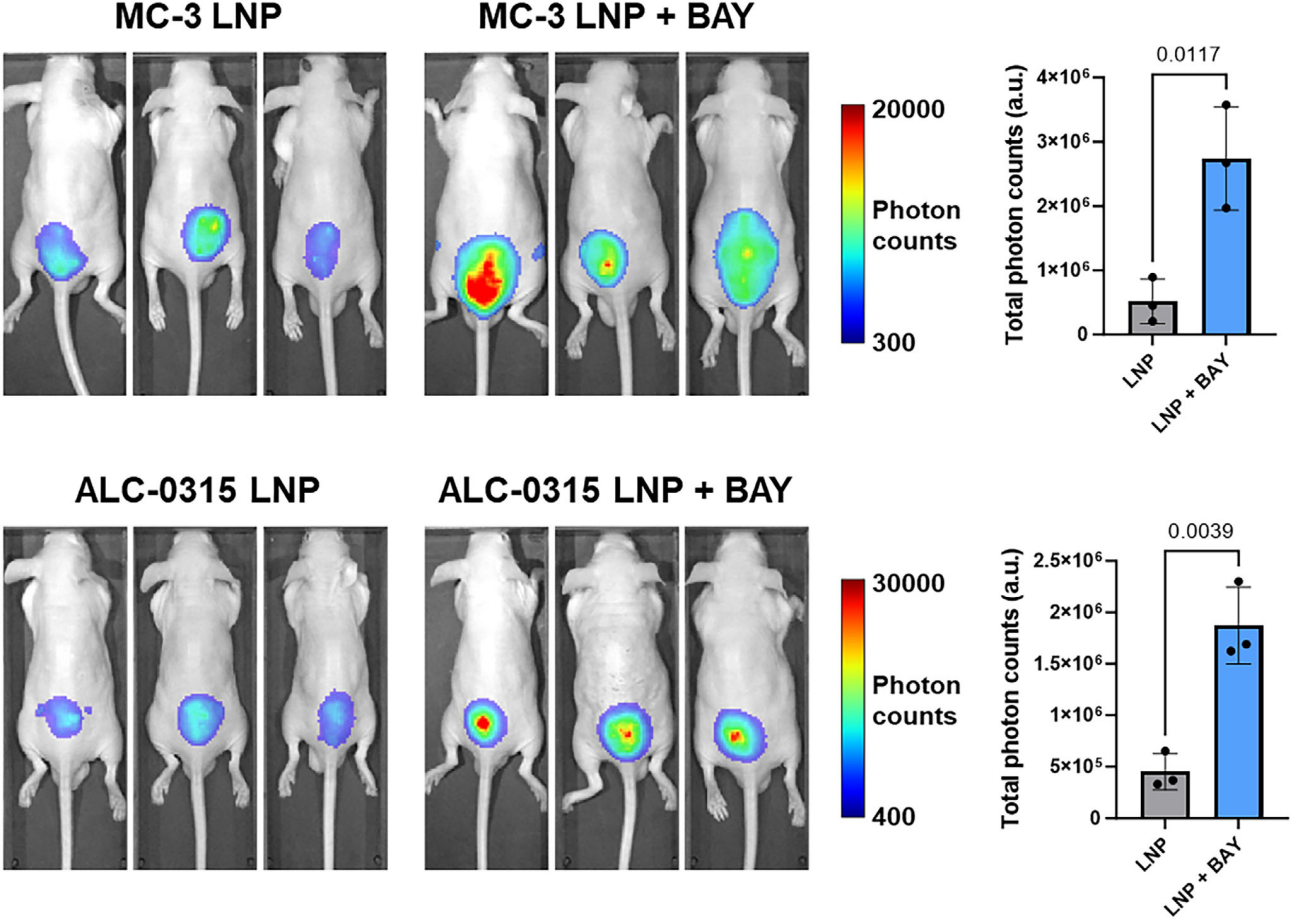
PIK3CA inhibition enhances the uptake of mRNA‐loaded LNPs following local administration. (*n* = 3, unpaired t‐test).

We first evaluated the pharmacokinetics and biodistribution of LNPs under PIK3CA inhibition using luciferase mRNA‐loaded LNPs formulated with MC‐3 and ALC‐0315 lipids. Mice were intraperitoneally (i.p.) administered 50 mg kg^−1^ BAY1082439 prior to LNP injection. The LNPs were then delivered either locally via subcutaneous injection (**Figure** **5**) or systemically via tail‐vein injection (Figure S12, Supporting Information). Notably, PIK3CA inhibition led to an overall increase in bioluminescent signal one‐day post–LNP administration, whereas the biodistribution pattern, as indicated by luciferase expression, remained largely unchanged.

**Figure 6:**
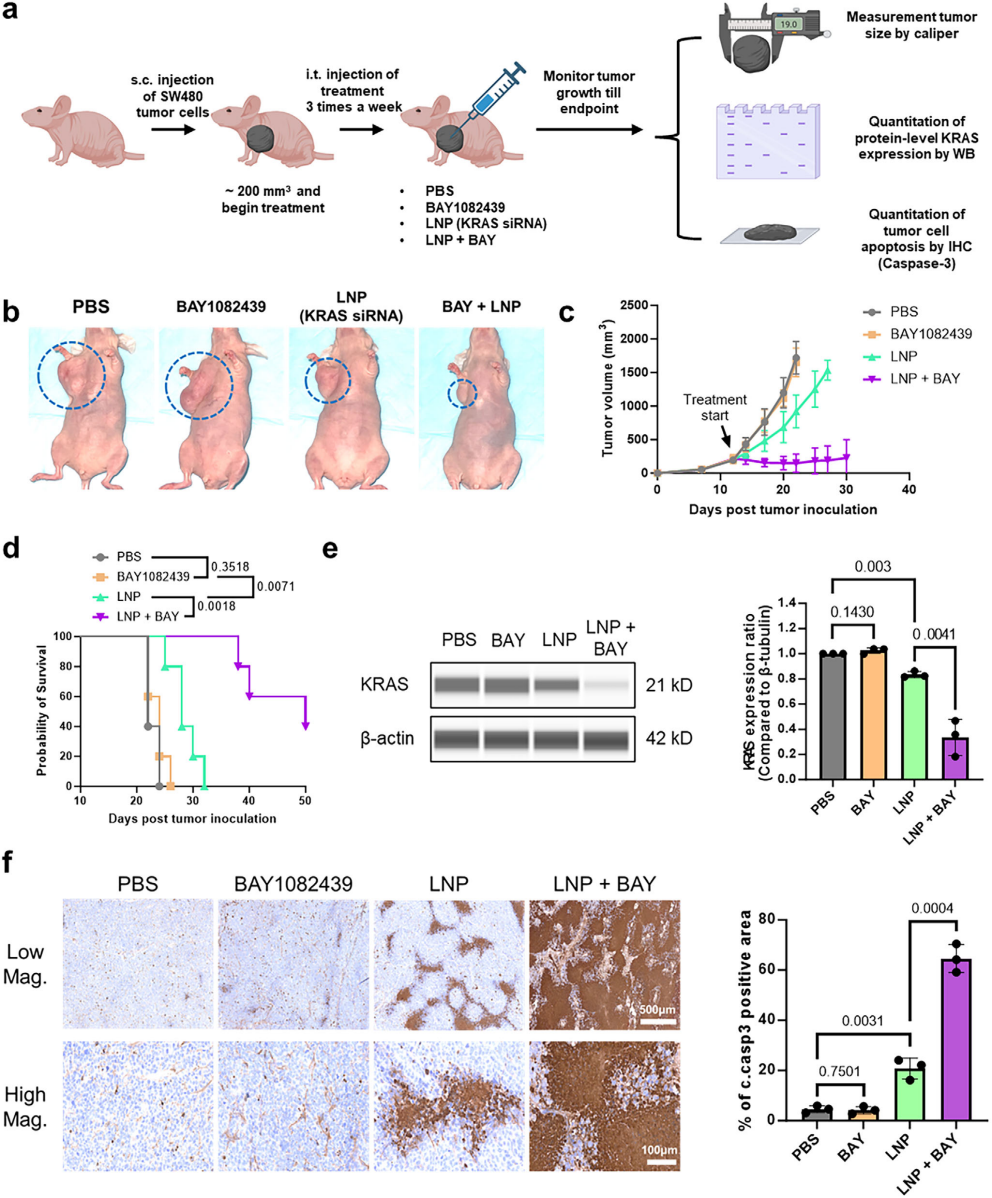
PIK3CA inhibition enhances the therapeutic efficacy of siRNA‐loaded LNPs in a mouse cancer model. A) Schematic of the SW480 xenograft mouse model used to assess in vivo therapeutic efficacy. SW480 colorectal cancer cells were subcutaneously implanted into nude mice. Once tumors reached an average volume of ≈200 mm^3^, mice were treated with siRNA‐loaded LNPs, BAY1082439, or their combination. LNPs were administered three times per week until the humane endpoint. Tumor progression was monitored, and tissues were harvested for molecular and histological analyses, including KRAS expression and apoptosis. B) Representative tumor images from day 20 post‐treatment initiation. Tumors from mice receiving combination therapy (BAY1082439 + LNPs) were markedly smaller than those from other treatment groups. C) Tumor growth kinetics of different treatment cohorts (*n* = 5). D) Median survival curves for each treatment group (*n* = 5, log‐rank test). E) Quantification of intratumoral KRAS protein expression levels (*n* = 3, unpaired t‐test). Combination therapy led to greater KRAS knockdown relative to LNP treatment alone. F) Quantification of intratumoral apoptosis based on immunohistochemistry (by cleaved caspase‐3, *n* = 3, unpaired t‐test).

Next, we established a SW480 xenograft model by subcutaneously injecting 5 × 10⁶ SW480 cells into nude mice. Once tumors reached 100–150 mm^3^, mice were randomly assigned to one of four treatment groups: (1) PBS (negative control); (2) BAY1082439 alone (50 mg kg^−1^, i.p.); (3) KRAS siRNA‐loaded LNPs (10 µg per mouse, intratumorally every two days); and (4) co‐administration of BAY and KRAS siRNA‐LNPs (**Figure** **6**a). Tumor volumes were measured every two days until the study endpoint.

The representative tumor images on day 20 are shown in Figure 6b and the quantitatively compared tumor volumes in Figure 6c. Tumors in the co‐administration group grew significantly more slowly than those in the BAY‐only or LNP‐only groups, suggesting a synergistic effect between the two treatments. This slowed tumor growth translated into a 100% increase in median survival compared to the PBS‐treated group (24 days vs 50 days, Figure 6d). To confirm the mechanism of action, we quantified KRAS protein expression using digital Western blotting (Figures 6e; S13, Supporting Information). The co‐administration group showed markedly reduced KRAS levels, indicating that BAY enhances the inhibitory effect of LNP‐delivered siRNA, likely by improving cellular uptake as demonstrated in our in vitro experiments (Figure 3d). Since KRAS is essential for the survival of SW480 cells,^[^ ^42^ ^]^ its reduced expression led to increased apoptosis (as indicated by cleaved caspase‐3 staining, Figure 6f). These results support the potential of BAY co‐administration as an effective strategy to improve the therapeutic efficacy of LNP‐based cancer treatments – a particularly attractive approach given BAY's compatibility with oral administration.

Beyond cancer therapy, LNP‐mediated delivery has shown promise in treating inflammatory diseases such as acute liver inflammation induced by acetaminophen (APAP) overdose^[^ ^43^ ^]^ and concanavalin A exposure.^[^ ^44^ ^]^ Since drug overdose remains the leading cause of acute liver failure in clinical settings,^[^ ^45^ ^]^ more effective therapeutic strategies is highly desired. However, current LNP‐based treatments for inflammatory hepatitis yield suboptimal outcomes, with substantial necrotic regions still evident following treatment.^[^ ^46^, ^47^ ^]^ This limitation prompted us to investigate whether our uptake‐enhancement strategy could be applied beyond malignant settings to benefit the treatment of inflammatory diseases.

**Figure 7:**
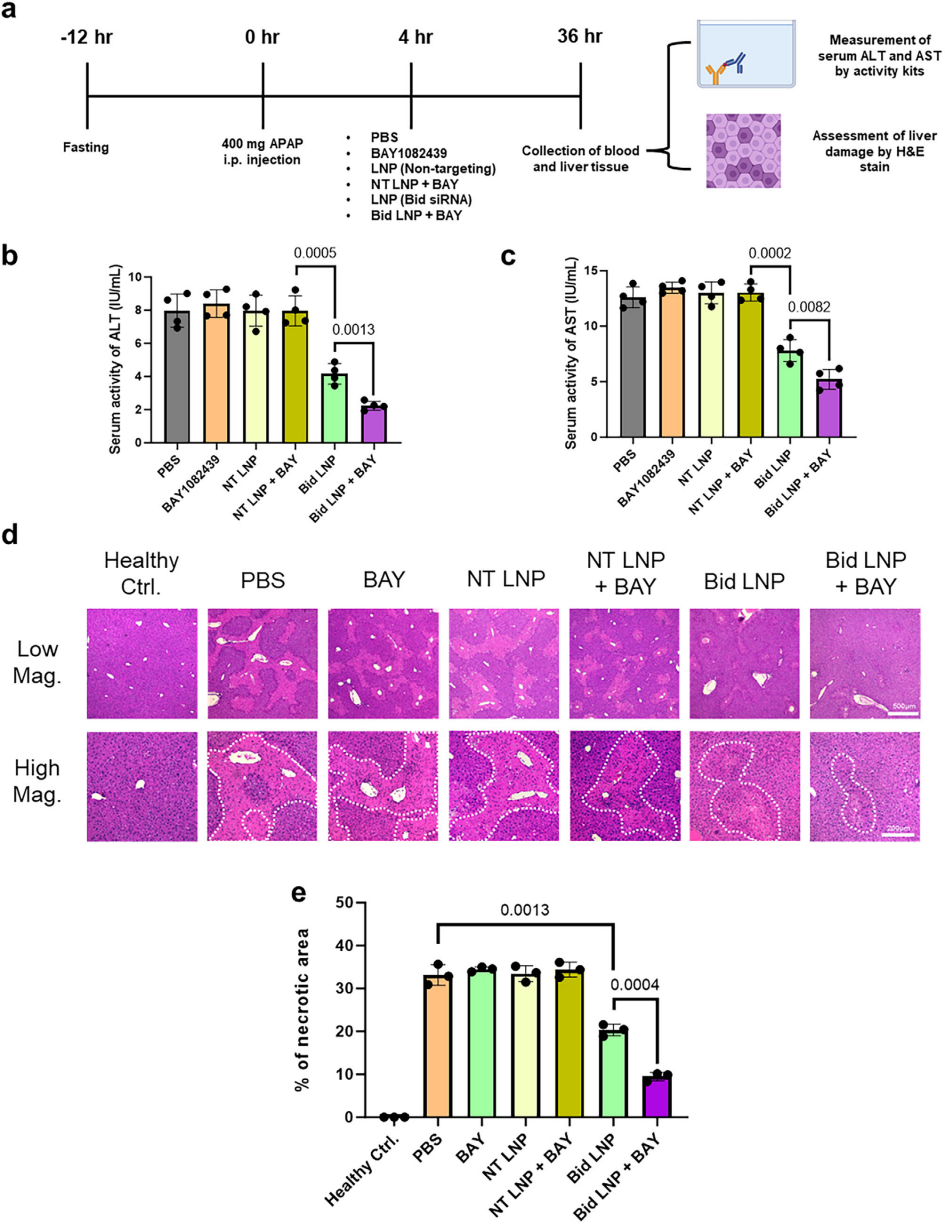
PIK3CA inhibition enhances the therapeutic efficacy of siRNA‐loaded LNPs in a mouse model of acute liver inflammation. A) Schematic of the APAP overdose‐induced liver inflammation model. Therapeutic intervention using non‐targeting siRNA‐loaded LNPs or Bid‐targeting siRNA‐loaded LNPs was administered with or without BAY1082439. Mice were euthanized 36 h after APAP administration for serum and tissue analysis. B) Serum alanine aminotransferase (ALT) activity was measured 36 h post‐treatment (*n* = 4, unpaired t‐test). Elevated ALT levels served as a biomarker for liver damage severity. C) Serum aspartate aminotransferase (AST) activity under the same treatment conditions (*n* = 4, unpaired t‐test). Elevated AST indicates hepatocellular injury. D) Representative H&E‐stained liver sections from each treatment group. Dashed white lines demarcate regions of hepatic tissue damage. E) Quantification of liver injury area based on histological analysis of H&E‐stained sections (*n* = 3, unpaired t‐test).

We induced acute liver inflammation in wild‐type C57BL/6J mice by intraperitoneally injecting 400 mg kg^−1^ of APAP following a 12‐h fasting period (**Figure** **7**a). Four hours after APAP administration, mice received retro‐orbital injections of either PBS, BAY, non‐targeting LNPs, a combination of non‐targeting LNPs and BAY, Bid siRNA‐loaded LNPs, or a combination of BAY and LNPs. At 36 h post‐APAP injection, the mice were euthanized, and blood and liver samples were collected to evaluate liver injury (Figure 7a). Liver damage was assessed through histological analysis and measurement of serum alanine aminotransferase (ALT) and aspartate aminotransferase (AST) levels – two of the most commonly used clinical biomarkers of liver injury.^[^ ^48^ ^]^

We found that co‐administering BAY with Bid siRNA‐loaded LNPs significantly reduced serum ALT and AST activities (Figure 7b,c), suggesting a lower degree of liver damage. Histological analysis further supported this finding (Figure 7d,e), as we observed minimal necrotic regions in the BAY + LNP‐treated group compared to the other groups. Together, these results indicate that BAY co‐administration enhances the therapeutic efficacy of LNP‐mediated siRNA treatment for inflammatory disease. Yet, in this study, we administered BAY intravenously to ensure rapid pharmacokinetics. Whether oral BAY administration can achieve similar therapeutic benefits in the context of acute inflammatory conditions warrants further investigation.

## 3 Discussion

Despite the clinical success of LNPs in delivering nucleic acids, their therapeutic efficacy remains limited by inefficient in vivo delivery – particularly at the level of cellular uptake.^[^ ^9^ ^]^ In this study, we report results from a genome‐wide CRISPR screen that systematically identifies genetic regulators of LNP uptake. By isolating both high‐ and low‐uptake cell populations, we captured a broader spectrum of genotype–phenotype relationships than previous studies, which focused primarily on uptake‐deficient cells.^[^ ^21^, ^22^, ^23^ ^]^ Our findings not only validate known endocytic and trafficking pathways essential for baseline delivery but also uncover gene knockouts that can enhance LNP uptake and potentially improve therapeutic efficacy through pharmacological targeting.

Among these newly identified genes, *PIK3CA* emerged as a particularly promising target thanks to its druggability. Using BAY1082439, a clinically evaluated PIK3CA inhibitor, we showed that pharmacological inhibition of PIK3CA enhances LNP uptake across multiple cell lines. This increased uptake led to improved siRNA‐induced gene silencing both in vitro and in vivo. These findings support the concept that selectively targeting host cell pathways can significantly improve nanoparticle delivery without the need to modify the particles’ biophysical or biochemical properties.

Our in vivo results further suggest that co‐administration of BAY1082439 with LNPs represents a broadly applicable translational strategy to enhance the therapeutic efficacy of nucleic acid‐based treatments for cancer and inflammatory diseases. Given BAY1082439's oral bioavailability and established pharmacokinetics, incorporating this co‐administration approach into future clinical trials should be straightforward. Moreover, the identification of other genes with higher NormZ scores than *PIK3CA* provides additional opportunities for developing selective inhibitors to further optimize delivery outcomes.

## 4 Conclusion

Taken together, our study underscores the power of integrating functional genomics with nanomedicine to uncover novel insights for drug delivery. By simultaneously tuning both particle and cellular parameters, we can move closer to realizing the full potential of precision nanomedicine.

## 5 Experimental Section

### Cell Culture

NCI‐H441 (RRID: CVCL_1561), SW480 (RRID: CVCL_0546), SW620 (RRID: CVCL_0547), MDA‐MB‐231 (RRID: CVCL_0062) were purchased from the American Type Culture Collection (ATCC) and cultured in RPMI‐1640 medium (22 400 121, Thermo Fisher) supplemented with 10% fetal bovine serum (FBS, Regular Grade, 35‐010‐CV, Corning) and 1% Penicillin‐Streptomycin (15 140 163, Thermo Fisher). Cells were passaged twice weekly by trypsinization to maintain confluency between 10% and 80%.

### Lipid Nanoparticles Synthesis

siRNA‐loaded lipid nanoparticles (LNPs) were prepared using the NanoGenerator Flex‐M system (PreciGenome) following a Patisiran‐like formulation. In brief, DLin‐MC3‐DMA (#34 364, Cayman Chemical), 1,2‐DSPC (#15 100, Cayman Chemical), Cholesterol (#57‐88‐5, Avanti Research), and C‐DMG‐PEG(2000, #37 149, Cayman Chemical) were mixed in the lipid molar ratio of 50:10:38.5:1.5 to form a Patisiran‐like formulation optimized for siRNA delivery.^[^ ^49^ ^]^ Negative control FAM‐ and Cy5‐labeled siRNAs were obtained from Sigma–Aldrich (SIC007 and SIC006, respectively). Pre‐designed siRNAs targeting KDM4A and KIF11 with a 1‐in‐3 guarantee were purchased from MedChemExpress (HY‐RS07213 for KDM4A, HY‐RS07255 for KIF11). siRNA sequences targeting mutant KRAS (referred as EFTX) and Bid were sourced from references [50] and [51] respectively, and synthesized as custom tube‐format oligonucleotides by Sigma–Aldrich.

### Lipid Nanoparticles Characterization

To assess the hydrodynamic diameter and polydispersity of synthesized LNPs, dynamic light scattering (DLS) measurements using the Zetasizer Nano ZSP (Malvern Panalytical) was performed. All measurements were carried out at 25 °C in disposable micro cuvettes (Malvern Panalytical) using a backscatter detection angle of 173°, following the manufacturer's recommended protocol. Briefly, freshly formulated LNPs were diluted 10× in DI water to a final RNA concentration of 0.1 µg µL^−1^. Diluted samples were gently vortexed and equilibrated for 2 min at room temperature before loading into the cuvette. Three technical replicates were measured per formulation, with each replicate consisting of five consecutive acquisitions (10 s per acquisition). Z‐average hydrodynamic diameter and polydispersity index (PDI) were automatically calculated using the instrument's built‐in software (ZS Xplorer v3.2).

### Lentiviral Production

The pooled plasmid of the Toronto KnockOut version 3 (TKOv3) CRISPR knockout library was obtained from Addgene (#90 294). Lentiviral particles were produced using Lenti‐X 293T cells (632 180, Takara Bio). Briefly, 8.0 µg of the TKOv3 plasmid was co‐transfected into 80% confluent Lenti‐X 293T cells along with 4.8 µg of psPAX2 (Addgene #12 260) and 3.2 µg of pMD2.G (Addgene #12 259) per 150 mm culture plate. The plasmid mixture (16.0 µg total in 50 µL of ddH2O) was added to 48 µL of X‐tremeGENE 9 DNA transfection reagent (XTG9‐RO, Roche), which had been preincubated for 5 min in 750 µL of Opti‐MEM (1 985 062, Thermo Fisher). The plasmid–transfection reagent mixture was incubated for an additional 25 min before being added dropwise to the cells. 18 h post‐transfection, the culture medium was replaced with fresh Dulbecco's Modified Eagle Medium (DMEM). Viral supernatant was harvested 48 h after the media change, snap‐frozen in liquid nitrogen, and stored at −80 °C until use.

### Pooled CRISPR Knockout Screen

A total of 200 million SW480 cells were infected with the prepared lentivirus at a pre‐determined multiplicity of infection (MOI) at 0.25–0.4 in the presence of 8 µg mL^−1^ polybrene (TR‐1003, Sigma–Aldrich) for 36 h. Infected cells were then selected with 5 µg mL^−1^ puromycin (#ant‐pr‐1, InvivoGen) for 72 h and maintained in regular culture medium (RPMI with 10% FBS and 1% penicillin/streptomycin) until further processing. Approximately 30% of the cells were harvested immediately after puromycin selection and stored as the time point 0 (T0) sample to assess initial library coverage.

Following 7–10 days of culture post‐selection, ≈ 500 million infected SW480 cells were treated with LNPs loaded with FAM‐labeled siRNA at a concentration of 1 µg mL^−1^ for 24 h. Cells were then sorted using a FACSMelody cell sorter (BD Bioscience) based on the intensity of FAM fluorescence. The top and bottom 5% of cell subpopulations were collected into separate tubes. Both sorted and unsorted cells from the same experiment day were stored at −80 °C prior to genomic DNA extraction.

Genomic DNA was extracted using the QIAamp Blood Midi or Maxi kits (#51 185 and #51 194, Qiagen). gRNA inserts were amplified in a two‐step PCR protocol using primers containing Illumina TruSeq adapters with i5 and i7 indices, as previously described.^[^ ^24^ ^]^ Final libraries were sequenced on an Illumina HiSeq2500 using a custom amplicon sequencing protocol. Briefly, sequencing included 21 initial cycles of base incorporation without imaging, followed by a 26‐base read that captured the gRNA sequence and included dual indexing (i7 followed by i5).

### Bioinformatics

Sample reads were trimmed by identifying the first 8 bp of the anchor sequences used in the barcoding primers and extracting the 20 bp immediately downstream of each anchor. Read counts from all samples in a screen were compiled into a matrix, and the percentage of recovered sgRNAs (defined as those with ≥1 raw read) was used as a quality control metric. Normalized Z scores (NormZ) and corresponding p‐values for sorted samples were calculated relative to same‐day unsorted controls using the drugZ algorithm.^[^ ^25^ ^]^ Pathway analysis was conducted using both overrepresentation analysis (ORA) and gene set enrichment analysis (GSEA). For GSEA, NormZ scores were treated as fold changes. Gene names, NormZ scores, and p‐values were uploaded to the easyGSEA web tool (https://tau.cmmt.ubc.ca/eVITTA/easyGSEA/) for pre‐ranked analysis using the built‐in database.^[^ ^52^ ^]^ For ORA, candidate hit genes were selected using a threshold of adjusted p‐value < 0.05 and NormZ score > 2. These gene lists were uploaded to Metascape (https://metascape.org) for pathway annotation using default settings.^[^ ^53^ ^]^ STRING analysis was performed via https://string‐db.org/ using the multiple protein inquiry function and organism “homo sapiens”.

### Flow Cytometry

LNP‐ and/or inhibitor‐treated cells were trypsinized and centrifuged to collect cell pellets. For samples treated with LNPs encapsulating FAM‐labeled siRNA, cell pellets were resuspended in 500 µL of phosphate‐buffered saline (PBS, 10 010 023, Thermo Fisher) containing 1% bovine serum albumin (BSA, A9418, Sigma–Aldrich), and immediately analyzed using an acoustic focusing flow cytometer (Attune CytPix, Thermo Fisher) equipped with a 4‐laser, 15‐channel configuration. A minimum of 20000 events was recorded per sample.

For Annexin V staining, cell pellets were resuspended in 100 µL of 1X Annexin V binding buffer (6592S, Cell Signaling Technology) containing 1 µL Annexin V‐FITC conjugate and 12.5 µL propidium iodide (PI) and incubated on ice for 10 mins in the dark. After staining, cells were diluted with 1X Annexin V binding buffer to a final volume of 250 µL and immediately analyzed on the Attune CytPix flow cytometer. Data were processed using FlowJo software (version 10.10.0, FlowJo LLC). A minimum of 20000 events was recorded per sample. Early apoptotic cells were defined as Annexin V+/PI‐, while necrotic cells were defined as Annexin V+/PI+.

### Fluorescence Microscopy

LNP‐ and/or inhibitor‐treated cells were incubated with FluoroBrite DMEM (A1896701, Thermo Fisher) and imaged using a Revolve R4 fluorescence microscope equipped with a 20X extra‐long working distance (ELWD) objective. Z‐stack images were acquired and processed using the maximum intensity projection function in Fiji (ImageJ; https://imagej.net/software/fiji/).^[^ ^54^ ^]^

### XTT Assays

XTT proliferation assays were performed according to the manufacturer's instructions (https://goldbio.com/product/4029/xtt‐sodium‐salt). In brief, XTT sodium salt was purchased from Gold Biotechnology and prepared by dissolving 5 mg of XTT in 4 mL of complete cell culture medium. Phenazine methosulfate (PMS, P9625, Sigma‐Aldrich) was dissolved in PBS at a concentration of 3 mg mL^−1^ to generate the PMS stock solution. For the detection solution, 10 µL of PMS stock was mixed with 4 mL of the freshly prepared XTT solution. Immediately after mixing, 100 µL of the detection solution was added to each well of a cell culture plate. Plates were incubated for 4 h at 37 °C with gentle shaking. Following incubation, absorbance was measured at 450 nm using a Varioskan plate reader (Thermo Fisher).

### Luciferase mRNA‐LNP Reporter Assay

To assess whether PIK3CA inhibition enhances mRNA delivery efficiency, commercially available luciferase (Luc) mRNA‐loaded LNPs were purchased from PackGene Biotech (Houston, USA). Two lipid formulations were tested: ALC‐0315 and SM‐102. In brief, SW480 cells were seeded in 96‐well plates at a density of 1 × 10^4^ cells per well and pretreated with BAY1082439 at a concentration of 15 nm (S8595, Selleck Chemicals, USA) for 24 h prior to LNP treatment. Cells were then incubated with Luc‐mRNA‐LNPs (150 ng mRNA per well) for an additional 24 h. Luminescence intensity was quantified using the Pierce Luciferase Assay System (16 177, Thermo Fisher) according to the manufacturer's instructions. Measurements were performed on a Varioskan plate reader (Thermo Fisher) with a 1 s integration time per well. Relative luminescence fold change was calculated by normalizing BAY‐treated samples to the LNP‐only samples.

### siRNA‐Based PIK3CA Knockdown

SW480 cells were seeded in 96‐well plates at 1 × 10^4^ cells per well and transfected with either siPIK3CA‐specific or non‐targeting control siRNA (HY‐RS07213 and HY‐RS00001, MedChemExpress, USA) at a final concentration of 50 nm using Lipofectamine RNAiMAX (13 778 075, Thermo Fisher). After 24 h of siRNA transfection, cells were washed twice with PBS, replaced with fresh complete medium, and incubated for an additional 2–4 h before LNP treatment. Cells were then treated with MC3‐Luc‐mRNA‐LNPs at 150 ng mRNA per well for 24 h. Luminescence intensity was measured using the Pierce Luciferase Assay System on a Varioskan plate reader. Relative luminescence was normalized to the LNP‐only control samples.

### BAY1082439 Dose–Response Assay

To determine the effective concentration of BAY1082439 for enhancing LNP‐mediated mRNA delivery, SW480 cells were seeded in 96‐well plates at 1 × 10^4^ cells per well and pretreated for 24 h with serial concentrations of BAY1082439 ranging from 0 to 100 nm (0, 0.1, 1, 3, 5, 10, 15, 20, and 40 nm). Cells were then treated with MC3‐Luc‐mRNA‐LNP at 150 ng mRNA per well in a 100 µL final volume and incubated for an additional 24 h. Luminescence intensity was measured using the Pierce Luciferase Assay System on a Varioskan plate reader. Relative luminescence was normalized to the LNP‐only control samples.

### Flow Cytometry Analysis of DiD–EGFP LNP Uptake and Expression

EGFP mRNA‐loaded lipid nanoparticles (EZNano DLin‐MC3‐DMA LNP) containing the fluorescent lipid tracer DiD were obtained from Helix Biotech (Knoxville, USA). SW480 cells were seeded in 12‐well plates at 2 × 10^5^ cells per well and pretreated with BAY1082439 or PBS for 24 h prior to LNP exposure. Cells were then incubated with the DiD–EGFP LNP (1 µg mRNA per well) for 24 h. After incubation, cells were washed twice with PBS, detached using Accutase (A1110501, Thermo Fisher), and resuspended in PBS containing 2% FBS. Flow cytometry was performed on an Attune CytPix flow cytometer (Thermo Fisher). DiD fluorescence was detected in the APC channel (excitation 640 nm, emission 670/30 nm), and EGFP fluorescence was detected in the FITC channel (excitation 488 nm, emission 530/30 nm). A minimum of 10000 events were collected per sample. Data were analyzed using FlowJo v10 (Tree Star, USA), and the mean fluorescence intensity (MFI) of DiD and EGFP was used to quantify nanoparticle uptake and mRNA expression, respectively.

### Animal Studies

All animal experiments were conducted in accordance with the protocol approved by the Charles River Accelerator and Development Lab (CRADL, 2024–2130 and 2025–2713). The following mouse strains were obtained from The Jackson Laboratory: C57BL/6J (RRID: IMSR_JAX:000664, male, 12–16 weeks), NU/J athymic nude (RRID: IMSR_JAX:0 02019, female, 16–24 weeks). All mice were housed in the CRADL Chicago facility under a controlled 12‐h light/dark cycle on a normal chow diet and provided water and libitum.

### In Vivo Imaging of Bioluminescence

MC3‐Luc‐mRNA LNPs and ALC‐0315‐Luc‐mRNA LNPs were used to evaluate LNP delivery efficiency and biodistribution in vivo. Female NU/J athymic nude mice were injected subcutaneously with 50 µL of LNP suspension containing 2 µg luciferase mRNA into the dorsal flank. For co‐treatment groups, BAY1082439 (50 mg kg^−1^) was administered intraperitoneally (i.p.) 4 h before LNP injection. 24 h after injection, mice received an intraperitoneal injection of D‐luciferin (150 mg kg^−1^, Gold Biotechnology, USA) 10 min prior to imaging. Bioluminescence was captured using an IVIS Luminina S5 system (PerkinElmer, USA). Quantification of photon flux was performed using Aura software (Spectral Instruments Imaging, USA). For biodistribution assessment, mice received tail‐vein injections of LNPs containing 5 µg luciferase mRNA in 100 µL PBS. BAY1082439 (50 mg kg^−1^) was i.p. administered 4 h prior to LNP injection. After 24 h, mice were injected with D‐luciferin (150 mg kg^−1^) and imaged as described above. Following in vivo imaging, mice were euthanized immediately, and major organs (liver, spleen, lung, heart, kidney) were collected for ex vivo bioluminescence imaging. Bioluminescence intensity from each organ was quantified using Aura software to assess tissue‐level LNP distribution.

### SW480 Tumor Xenograft Model

A total of 5 × 10^6^ SW480 cells were xenografted into the axillary region of female nude mice. Tumor growth was monitored every two days using caliper measurements and calculated using the modified ellipsoid formula: tumor volume = 0.5 × (length × width^2^). On day 12, the average tumor volume reached 150–200 mm^3^, and treatment was initiated. One of the following treatments was administered intratumorally every two days until a humane endpoint was reached, unless otherwise specified: PBS, BAY1082439 (50 mg kg^−1^), LNP encapsulating KRAS siRNA (10 µg), or a combination of BAY1082439 and LNP. On day 20, a subset of mice from each treatment group was euthanized for tumor collection.

Portions of collected tumor tissue were fixed in 10% neutral‐buffered formalin (NBF; HT501128, Sigma‐Aldrich) for one week and submitted to the Northwestern Mouse Histology & Phenotyping Laboratory (MHPL) (https://www.feinberg.northwestern.edu/sites/mhpl/) for immunohistochemical staining. Slides were stained for cleaved Caspase‐3 (1:400 dilution; 9664S, Cell Signaling Technology). Detection was performed using an anti‐rabbit HRP‐conjugated polymer (MACH2, MHRP520, Biocare Medical, Pacheco, CA) and visualized with 3,3′‐diaminobenzidine (DAB; Vector Labs, Newark, CA). Slides were counterstained with hematoxylin and imaged under a Revolve R4 microscope at 10X magnification.

Additional tumor tissues were homogenized on ice using a Bead Mill 24 Homogenizer (Thermo Fisher Scientific), and total proteins were extracted using RIPA lysis buffer supplemented with protease and phosphatase inhibitors (89 900,78 440, Thermo Fisher). The homogenates were centrifuged at 14000 × g for 15 min at 4 °C to remove debris, and the supernatants were collected for downstream analysis. Protein concentrations were quantified using a BCA assay (23 227, Thermo Fisher) following the manufacturer's instructions. Western blot analysis was performed using an automated capillary‐based system (JESS, ProteinSimple, Bio‐Techne). Protein lysates were diluted to a final concentration of 0.5 µg µL^−1^ in 0.1X sample buffer, and 3 µL of each sample was loaded per well. Primary antibodies used were monoclonal rabbit IgG anti‐KRAS‐G12V (14 412, Cell Signaling Technology) and polyclonal rabbit IgG anti‐β‐Actin (20536‐1‐AP, ProteinTech). IgG anti‐KRAS was diluted at 1:50 in milk‐free antibody diluent (SM‐W004, ProteinSimple) while IgG anti‐β Actin was dilute at 1:1000. Antibodies were added at 5 µL per well. HRP‐conjugated secondary antibody and enhanced chemiluminescence (ECL) reagents (DM001, ProteinSimple) were used for signal detection according to the manufacturer's protocol. All reagents and capillaries were from the 12 to 230 kDa separation module (SM‐W004, ProteinSimple). Data acquisition and analysis were performed using Compass for Simple Western software. High dynamic range (HDR) 4.0 imaging mode was applied with automatic peak identification. Both peak height and area were quantified. Exposure time was set to 16 s for KRAS and 2 s for β‐Actin.

### Acetaminophen Overdose Model

The acetaminophen (APAP) overdose model was performed using male C57BL/6J mice. Pharmaceutical secondary standard‐grade APAP (PHR1005, Sigma–Aldrich) was dissolved in PBS at 56 °C to a final concentration of 20 mg mL^−1^ and stored at room temperature until use. All mice were fasted 12 h before APAP injection to standardize liver metabolic activity.

APAP was administered via intraperitoneal injection at a dose of 400 mg kg^−1^. After injection, mice were maintained on a normal chow diet with water provided ad libitum. Four hours post APAP injection, mice received a retro‐orbital injection of one of four treatment conditions: PBS, BAY1082439 (50 mg kg^−1^), LNP encapsulating control siRNA (10 µg), LNP encapsulating Bid siRNA (10 µg), or a combination of BAY1082439 and Bid siRNA LNP or control siRNA‐LNP.

36 h post‐APAP injection, mice were euthanized for the collection of liver tissue and blood samples. Whole blood was collected in serum separator tubes (02‐683‐97B, Fisher Scientific), left undisturbed for 15 min to allow clotting, and then centrifuged at 15000 × g for 15 min. The resulting serum was transferred to 1.5 mL microcentrifuge tubes for downstream analysis.

Liver samples were fixed in 10% neutral‐buffered formalin (NBF; HT501128, Sigma–Aldrich) for one week, then submitted to the Northwestern MHPL for hematoxylin and eosin (H&E) staining. Serum levels of alanine transaminase (ALT, 700 260, Cayman Chemical) and aspartate aminotransferase (AST, 701 640, Cayman Chemical) were measured according to the manufacturer's protocols. Briefly, 150 µL of substrate, 20 µL of cofactor, and 20 µL of serum were added to each well of a 96‐well plate and incubated at 37 °C for 15 min. Reactions were initiated by adding 20 µL of initiator. Absorbance at 340 nm was recorded immediately and measured every minute for 30 min at 37 °C using a plate reader. Enzyme activity was determined based on the rate of change in absorbance over time.

### Statistical Analysis

All statistical analyses were performed using Prism GraphPad (v10.1.2, GraphPad Software). Results are presented as mean ± standard deviation unless stated otherwise. Each data point represents a biological replicate unless specified differently. P‐values were calculated using the built‐in unpaired t‐test analysis functions of Prism GraphPad unless specified differently.

## Conflict of Interest

The authors declare no conflict of interest.

## Supporting information



Supporting Information

Supporting Information

## Data Availability

The data that support the findings of this study are available in the supplementary material of this article.
