# Supplementary material for: Genome‐Wide CRISPR Screen Reveals PIK3CA Inhibition Enhances Lipid Nanoparticle‐Mediated siRNA Delivery
Source: Adv Sci (Weinh). 2025 Dec 7;13(11):e17617. doi: 10.1002/advs.202517617 (PMC12931213; doi:10.1002/advs.202517617)
Supplement: Supplementary file 1 — Supporting Information [file ADVS-13-e17617-s002.docx]

Supporting Information

Genome-wide CRISPR Screen Reveals PIK3CA Inhibition Enhances Lipid Nanoparticle-mediated siRNA Delivery

*Wenhan Wang^1,2^, Kangfu Chen^2,3^, and Zongjie Wang^1,2,*^*

^1^Chan Zuckerberg Biohub Chicago, Chicago, IL, 60607, USA

^2^Department of Biomedical Engineering, McCormick School of Engineering, Northwestern University, Evanston, IL, 60208, USA

^3^Present Address: School of Integrated Circuits and Electronics, Beijing Institute of Technology, Beijing, 100081, P.R. China

*Corresponding Author: Zongjie Wang, Email: [daniel.wang@czbiohub.org](mailto:daniel.wang@czbiohub.org)

**Table of Contents**

**Supporting Figures Page S2**

**Supplementary Figures**


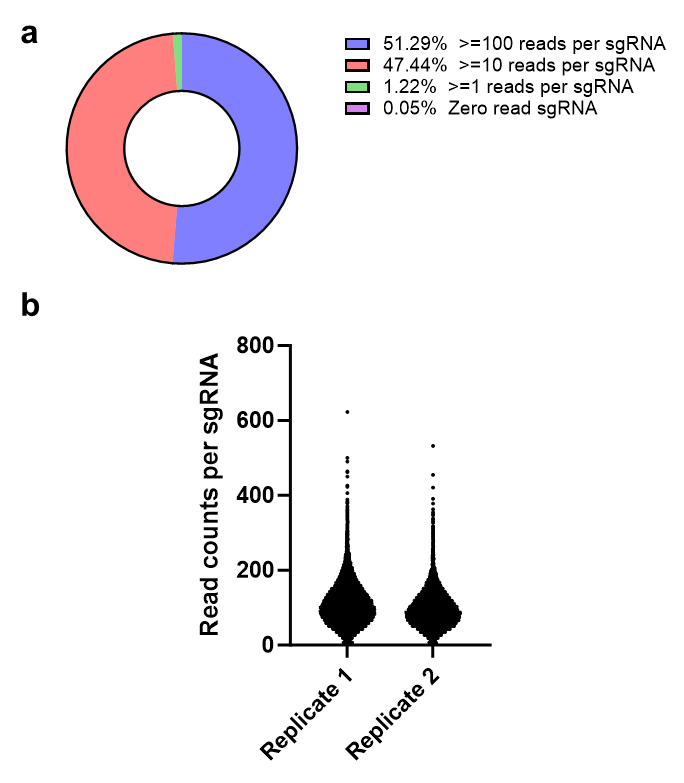


**Figure. S1 Quality assessment of the prepared CRISPR libraries.** (a) Distribution of sgRNAs following transduction and puromycin selection. (b) Comparison of sgRNA counts between two biological replicates.

**Figure. S2 Quality assessment of synthesized LNPs.** Hydrodynamic diameter and polydispersity index (PDI) of LNPs were measured using dynamic light scattering (DLS). The synthesized LNPs exhibited uniform size distribution and low PDI values, indicating a monodisperse nanoparticle population.


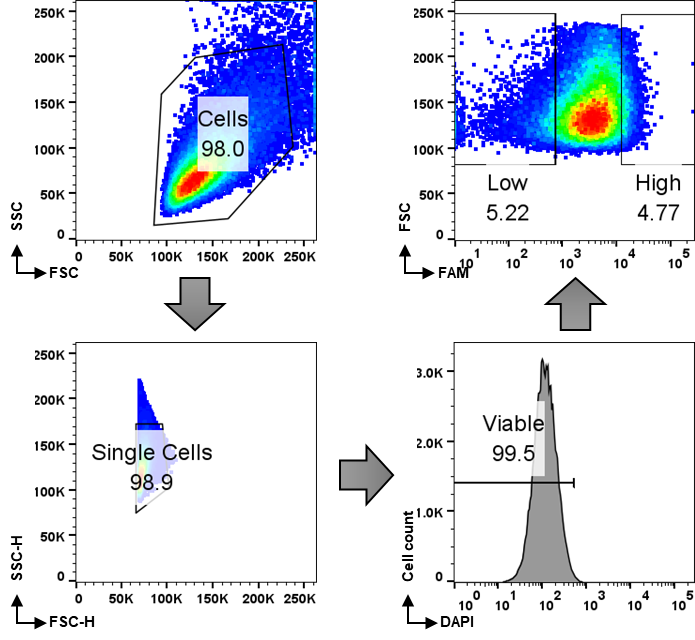


**Figure. S3 Representative gating strategy used for FACS.**


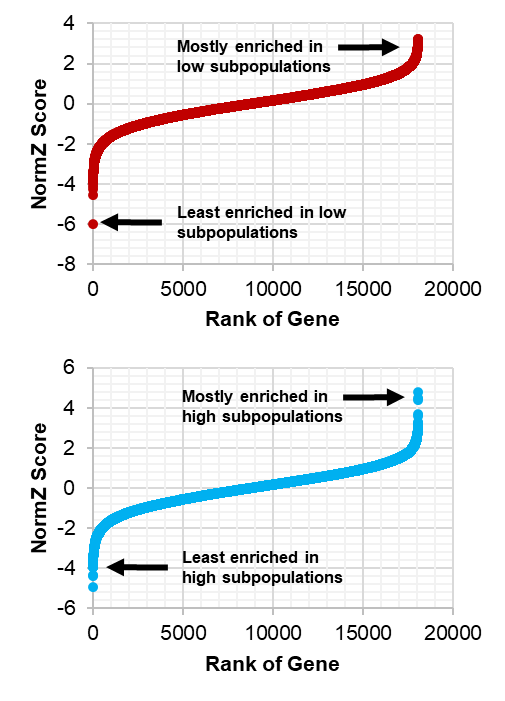


**Figure. S4 Results of DrugZ analysis identifying gene hits from CRISPR screen.** Detailed of the top-ranked genes are discussed in the main manuscript.


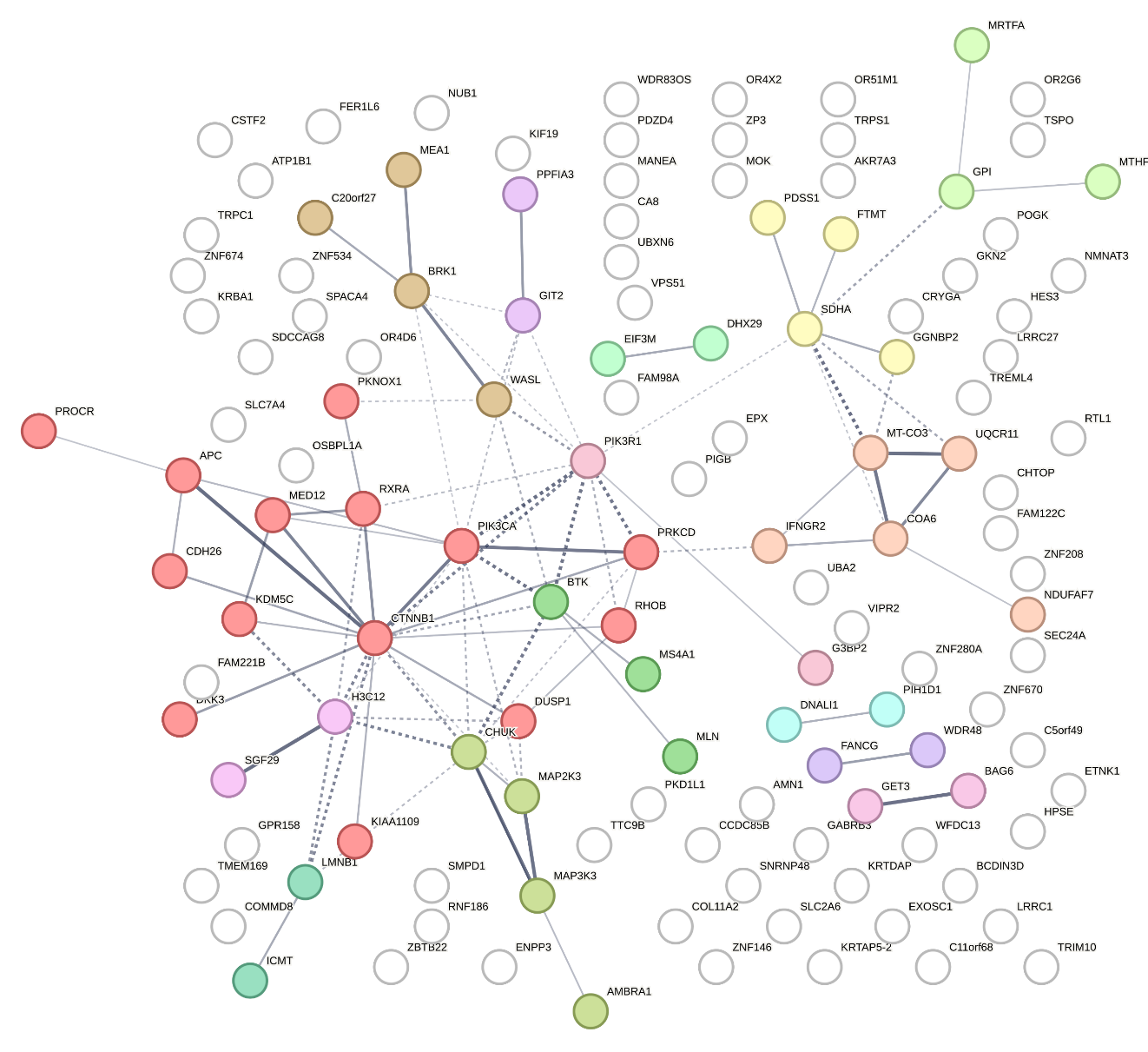


**Figure. S5 STRING analysis reveals *CTNNB1* and *PIK3CA*’s central role in the hits (p < 0.01) from uptake improving genes.**


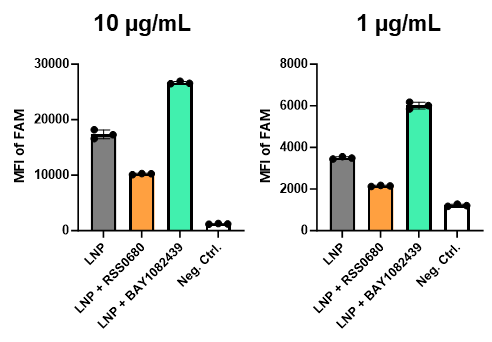


**Figure. S6 Quantitation of MFI of FAM under different treatment conditions (n = 3).**

**
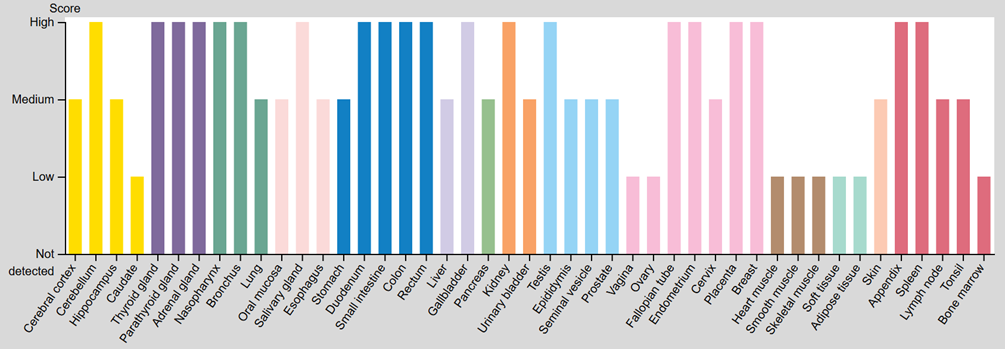
**

**Figure. S7 Protein-level expression of PIK3CA across various tissue types.** Data indicates that PIK3CA is broadly expressed across the majority of examined organs, suggesting its potential as a broadly applicable therapeutic target. Source: Protein Atlas, <https://www.proteinatlas.org/ENSG00000121879-PIK3CA/tissue>


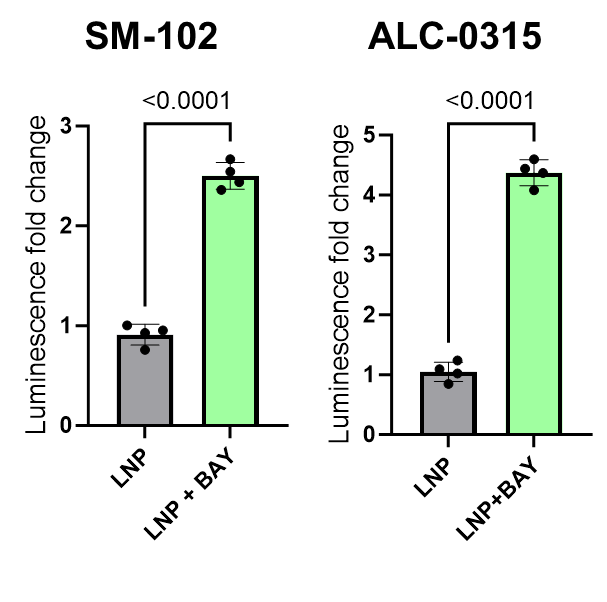


**Figure. S8 PIK3CA inhibition enhances the uptake of mRNA-loaded LNPs formulated with SM-102 or ALC-0315 lipids.** (n = 4, unpaired t-test)

**Figure. S9 siRNA-mediated PIK3CA knockdown confirms that the inhibitory effects of BAY are specifically due to PIK3CA inhibition rather than off-target effects.** (n = 4, unpaired t-test)

**Figure. S10 Investigation of the dose-dependent effects of BAY on the promotion of LNP uptake.**

**
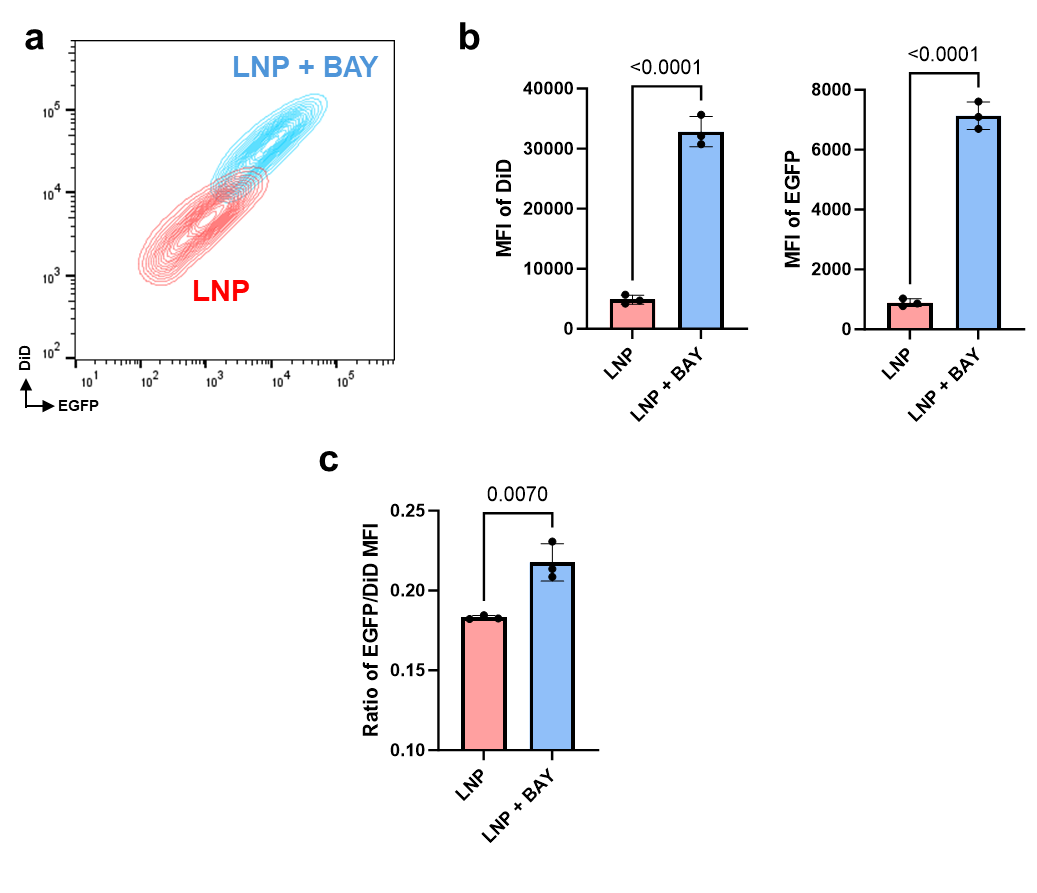
**

**Figure. S11 PIK3CA inhibition primarily enhances LNP endocytosis.** (a) Flow cytometric analysis of DiD (LNP uptake) and EGFP (mRNA expression) signals in SW480 cells treated with or without BAY. (b) Quantification of median fluorescence intensity (MFI) for DiD and EGFP. (c) Ratio of EGFP to Di signals. (n = 3, unpaired t-test)

**
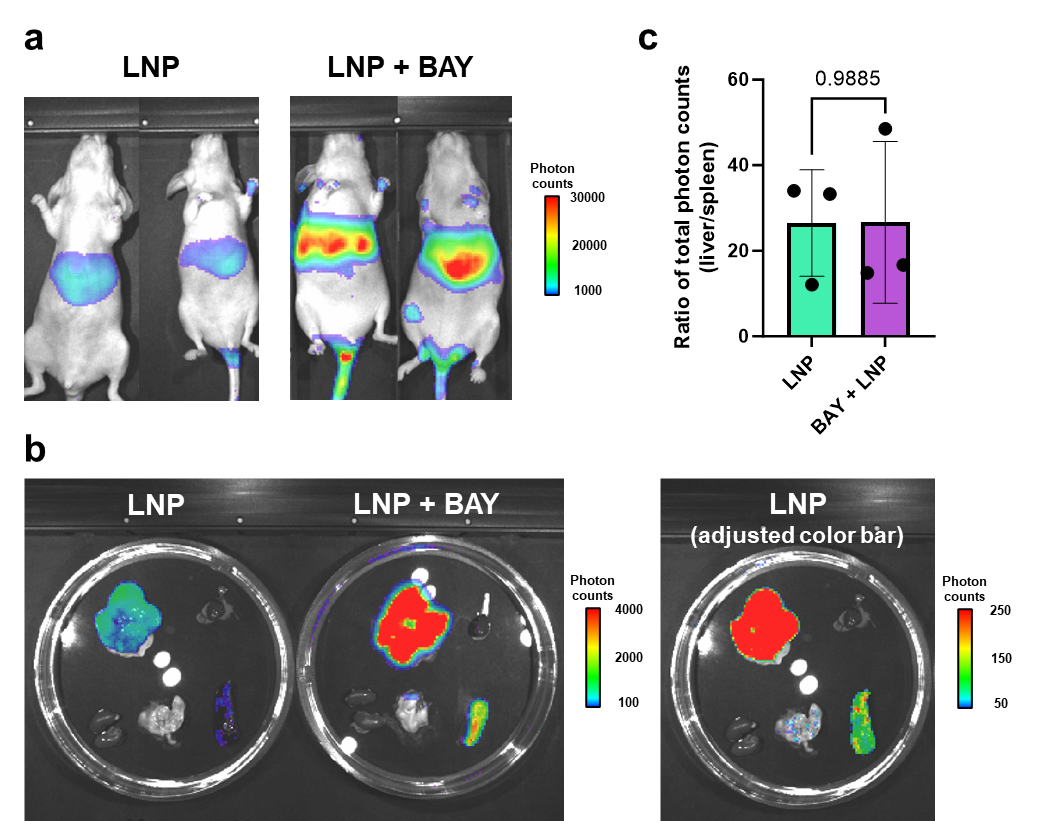
**

**Figure. S12 PIK3CA inhibition enhances the uptake of mRNA-loaded MC-3 LNPs following systemic administration.** (a) Representative IVIS images showing total photon counts in live animals with or without BAY treatment. (b) Representative IVIS images showing organ-level biodistribution of LNPs with or without BAY treatment. (c) Quantification of the liver-to-spleen photon count ratio. (n = 3, unpaired t-test)


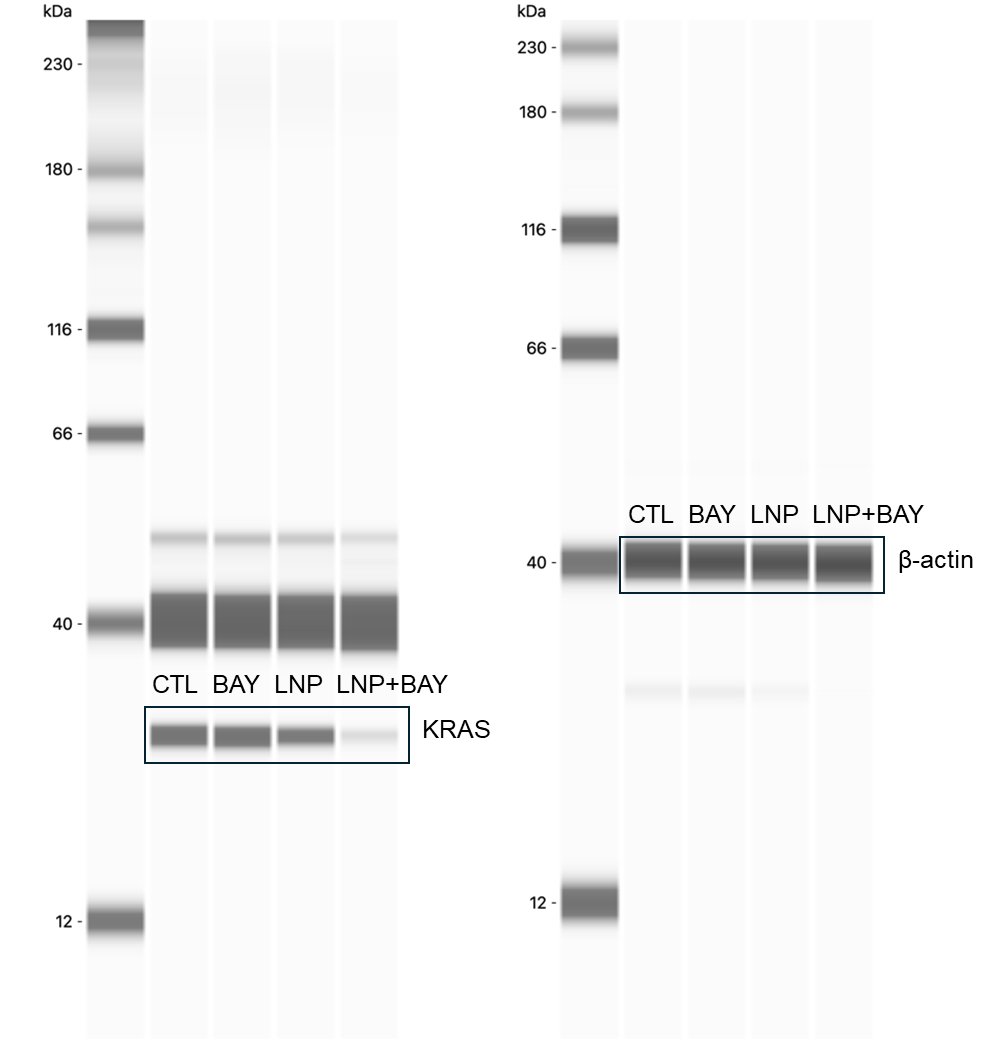


**Figure. S13 Uncropped gel images from the automated western blot system.** Representative Western blot images showing KRAS (exposure time: 16 s) and β‑actin (exposure time: 2 s) detection.
